# MedSegNet10: A Publicly Accessible Network Repository for Split Federated Medical Image Segmentation

**DOI:** 10.3390/bioengineering13010104

**Published:** 2026-01-15

**Authors:** Chamani Shiranthika, Zahra Hafezi Kafshgari, Hadi Hadizadeh, Parvaneh Saeedi

**Affiliations:** School of Engineering Science, Simon Fraser University, Burnaby, BC V5A 1S6, Canada; hadi_hadizadeh@sfu.ca (H.H.); parvaneh_saeedi@sfu.ca (P.S.)

**Keywords:** distributed learning, diverse network architectures, medical image segmentation, open source repository, split federated learning

## Abstract

Machine Learning (ML) and Deep Learning (DL) have shown significant promise in healthcare, particularly in medical image segmentation, which is crucial for accurate disease diagnosis and treatment planning. Despite their potential, challenges such as data privacy concerns, limited annotated data, and inadequate training data persist. Decentralized learning approaches such as federated learning (FL), split learning (SL), and split federated learning (SplitFed/SFL) address these issues effectively. This paper introduces “MedSegNet10,” a publicly accessible repository designed for medical image segmentation using split-federated learning. MedSegNet10 provides a collection of pre-trained neural network architectures optimized for various medical image types, including microscopic images of human blastocysts, dermatoscopic images of skin lesions, and endoscopic images of lesions, polyps, and ulcers. MedSegNet10 implements SplitFed versions of ten established segmentation architectures, enabling collaborative training without centralizing raw data and labels, reducing the computational load required at client sites. This repository supports researchers, practitioners, trainees, and data scientists, aiming to advance medical image segmentation while maintaining patient data privacy.

## 1. Introduction

Medical image segmentation plays a crucial role in the healthcare sector, serving as a foundational task for precise diagnosis, treatment planning, and patient care [1,2]. The emergence of decentralized training approaches, such as federated learning (FL) [3], split learning (SL) [4], and split federated learning (SplitFed learning/SFL) [5], has opened up new possibilities for collaborative, privacy-preserving, and resource-constrained model training across hospitals, research institutes, and healthcare organizations. Despite the considerable potential of decentralized learning approaches, their implementation for medical image segmentation still faces several practical challenges. One notable obstacle is the limited availability of well-designed and rigorously evaluated deep learning networks that can be readily adapted to SplitFed training pipelines. Addressing this gap requires accessible, standardized, and benchmarked resources that allow researchers to support reproducible experimentation and facilitate fair architectural comparison.

Alongside these technical challenges, interpretability has become a parallel requirement for the successful deployment of AI systems in real clinical environments. Clinicians expect models not only to perform accurately but also to provide insights that are consistent with clinical reasoning. Meta-analyses such as [6,7] underscore that transparency, usability, and clinician-aligned explanations are crucial for trustworthy AI-assisted decision support. This growing emphasis on interpretability poses additional challenges for decentralized learning: if medical image segmentation models are to be trained collaboratively across institutions, they must also provide insight into how decisions are made, particularly in high-stakes medical settings.

In this paper, we introduce the first documented repository of SplitFed networks—MedSegNet10 tailored specifically for medical image segmentation tasks. Aligned with the recent evolution of semantic segmentation networks, we curate ten top-performing models based on their demonstrated effectiveness in medical imaging [8,9]. These include UNet [10], SegNet [11], DeepLabV3 [12], DeepLabV3+ [13], RefineNet [14], CGNet [15], SUNet [16], DUCK-Net [17], Attention UNet [18], and Swin-UNet [19]. We train and evaluate all SplitFed variants using three datasets with distinct imaging characteristics: Human Against Machine (HAM10K) [20], KVASIR-SEG [21], and our proprietary Blastocysts dataset [22]. Ultimately, the repository aims to provide a well-structured, reproducible SplitFed benchmark that future work can expand to additional imaging domains and that can be enriched with interpretability-oriented components in subsequent research.

The primary objective of this work is to develop and release a publicly accessible repository of SplitFed-ready medical image segmentation networks. To this end, we standardize, benchmark, and streamline SplitFed development by rigorously implementing and faithfully reproducing SplitFed variants of ten well-established segmentation models. MedSegNet10 is designed as a practical and comprehensive resource, providing both novice and experienced practitioners with a unified platform for implementing and evaluating SplitFed-based segmentation. It integrates multiple architectures within a single framework, enabling systematic and rigorous comparisons of structural properties, performance characteristics, and SplitFed behaviour across models. Rather than introducing new SplitFed algorithms or novel segmentation architectures, this work focuses on establishing the first unified, reusable, and reproducible SplitFed repository for medical image segmentation. All included networks adhere to standardized split-point definitions, consistent client–server partitioning, and harmonized training pipelines. By creating this consistent experimental foundation, MedSegNet10 addresses a critical gap in the literature and provides essential infrastructure for rigorous cross-architecture comparison and future SplitFed research.

The structure of this paper is as follows. Section 2 provides an overview of SplitFed fundamentals and the current state-of-the-art in SplitFed for medical image segmentation, including its integration with emerging transformer-based architectures. It also reviews publicly available federated network repositories, outlines key concepts in semantic segmentation, and describes the architectures of our implemented split models, concluding with a discussion of our split-point selection strategy. Section 3 presents the experimental setup, results, and evaluation. Section 4 discusses the current limitations of MedSegNet10 and outlines potential future directions. Finally, Section 5 concludes the paper.

## 2. Related Works

This section reviews related work on SplitFed learning, publicly available federated repositories, and semantic segmentation, including notable semantic segmentation networks. Also, it offers insights into our decisions on split point selection for designing our split networks.

### 2.1. Split Federated Learning (SplitFed)

The technical evolution of SplitFed research reflects a progression from foundational feasibility studies toward more specialized problem domains. The initial SplitFed work [5] established the core client–server partitioning mechanism and demonstrated its advantages in privacy preservation achieved via FL and reduced client workload achieved via SL. However, it did not address heterogeneous data distributions or noisy communication channels. Subsequent studies targeted specific weaknesses of the original formulation: SplitAvg [23] introduced heterogeneity-aware aggregation to improve generalization on non-IID data, Dynamic corrected SplitFed [24] incorporated homomorphic encryption to mitigate security risks, and Quality-Adaptive SplitFed [25] addressed label imperfections—a recurring challenge in medical datasets. Smart SplitFed [26] further tackled robustness under unstable or noisy communication, a practical issue in multi-hospital deployments. More recently, transformer-based SplitFed extensions [27,28,29,30] explored architectures capable of capturing long-range dependencies, demonstrating improved performance in high-dimensional medical imaging tasks but at the cost of increased communication overhead and memory usage on the server. The studies [30,31,32] highlight the effectiveness of SplitFed transformers used in medical imaging tasks.

Despite this progression, prior studies remain isolated in scope: each investigates a single architecture under a narrow set of conditions, with no standardization of split-point selections, model partitioning strategies, or training pipelines across architectures. As a result, direct comparison among SplitFed variants is currently difficult, and the field lacks a unified benchmark for evaluating model behaviour across diverse network architectures and medical imaging domains. This fragmentation motivates the development of MedSegNet10, which consolidates these design elements into a consistent and reproducible SplitFed framework.

**Figure 1:**
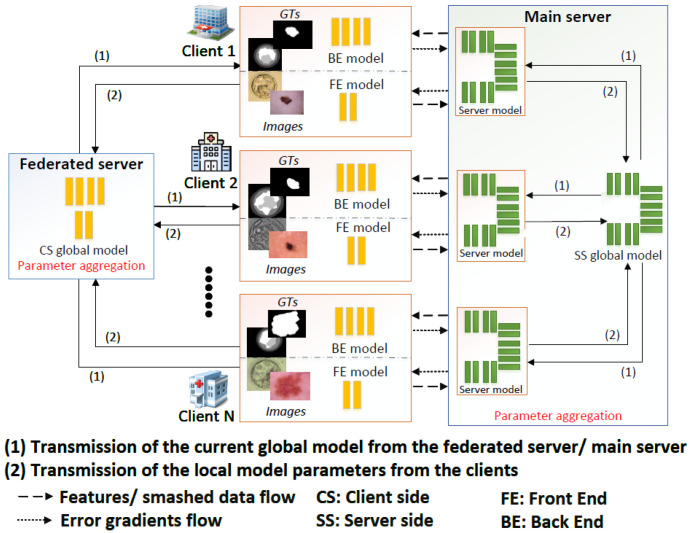
SplitFed architecture with multiple clients [5], where each client uses the split UNet as its model [33].

Figure 1 illustrates our SplitFed architecture, considering each client’s network backbone as the UNet. In this U-shaped architecture, each client has two split points: the front-end (FE) and back-end (BE) sub-models, with the server (SS) sub-model in between. The FE sub-model exclusively accesses the images, while the BE sub-model solely interacts with the ground truth (GT) data. Both the FE and BE sub-models constitute a small portion of the UNet architecture and are located on the client side, while the server sub-model, containing the majority of the layers, handles the main computational burden of training. This splitting process was consistently applied across all ten selected models.

### 2.2. Publicly Available Federated Repositories

Various public repositories for federated learning (FL), particularly those with annotated medical images [33], alongside numerous federated data analysis programs [34,35,36], are available for public access. Notable federated network repositories include TensorFlow Federated [37], Google Federated Research [38], LEAF [39], Fedmint [40], Federated Scope [41], FedLab [42], FATE [43], FedIIC [44], QuickSQL [45], GraphQL-Mesh [46], MedAugment [47], OpenMined’s PyGrid [48], FedCT [49], and COALA [50]. Among these, FATE, MedAugment, OpenMined’s PyGrid, and FedCT are specifically designed for medical imaging tasks.

Although these repositories provide valuable tools for federated experimentation, they primarily focus on general-purpose FL pipelines, data-sharing frameworks, or isolated model implementations. Existing platforms do not provide a unified, reusable collection of *SplitFed* architectures for medical image segmentation, and they do not standardize split-point definitions, client-server model partitioning, or consistent training pipelines across multiple segmentation networks. Prior SplitFed studies typically evaluate a single model or a narrow experimental setup, limiting reproducibility and cross-architecture comparison. These limitations reveal the need for a robust and adaptable SplitFed network repository that supports collaborative and reproducible research. MedSegNet10 directly addresses this gap by streamlining the implementation, training, and evaluation of SplitFed architectures in medical image segmentation.

### 2.3. Image Segmentation Models

Image segmentation is essential in healthcare for the accurate identification and extraction of regions within medical images, which is critical for disease detection, diagnosis, and treatment planning [8]. Enhancing the capability of algorithms to analyze complex medical data plays a vital role in the interpretation of medical images. Deep learning applications, such as SplitFed, further emphasize the significance of image segmentation by managing large-scale data across institutions while preserving privacy, thereby improving accuracy and efficiency. Semantic segmentation takes this further by classifying each pixel within identified regions, such as tumors or organs, providing detailed representations crucial for precise diagnosis and treatment. This section highlights MedSegNet10’s contributions to advancing semantic segmentation in medical imaging, addressing its complexities, and enhancing image content understanding.

**Figure 2:**
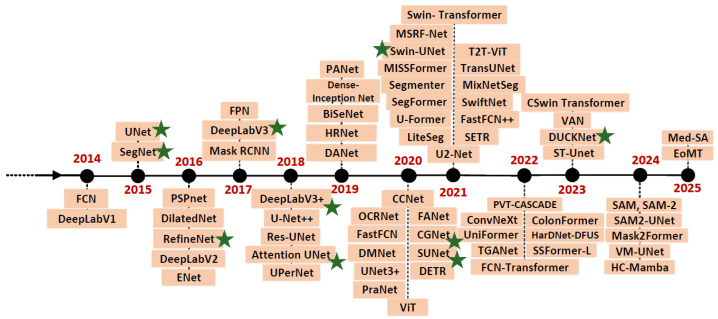
Semantic segmentation networks’ development timeline. Networks marked with green stars are those included in our work.

Figure 2 illustrates a timeline of prominent semantic segmentation networks that have emerged in recent decades, with their full names and corresponding references listed in the order shown in the figure. These include:

1. FCN [51]: Fully Connected Network.
2. UNet [10]: UNetwork.
3. SegNet [11]: Segmentation Network.
4. PSPnet [52]: Pyramid Scene Parsing Network.
5. ENet [53]: Efficient Neural Network.
6. RefineNet: Refining Segmentation-based Network.
7. DeepLab [12,13,54,55]: Deep Labelling for Semantic Image Segmentation.
8. Attention UNet: Attention-based UNet.
9. FPN [56]: Feature Pyramid Network.
10. Mask RCNN [57]: Mask Region-based Convolutional Neural Network.
11. PANet [58]: Path Aggregation Network.
12. BiseNet [59]: Bilateral Segmentation Network.
13. HRNet [60]: High-Resolution Network.
14. OCRNet [61]: Object-Contextual Representations for Semantic Segmentation.
15. DANet [62]: Dual Attention Network.
16. CCNet [63]: Criss-Cross Attention Network.
17. SETR [64]: Spatially Enhanced Transformer.
18. UPerNet [65]: Unified Perceptual Parsing Network.
19. FastFCN [66]: Fast Fully Convolutional Network.
20. SUNet [16]: Strong UNet.
21. FANet [67]: Feature Aggregation Network.
22. DMNet [68]: Dense Multi-scale Network.
23. CGNet [15]: Context-Guided Network.
24. DETR [69]: DEtection Transformer.
25. PraNet [70]: Parallel Reverse Attention Network.
26. ViT [71]: Vision Transformer.
27. Swin-UNet: Swin Transformer-based UNet.
28. MSRF-Net [72]: Multi-Scale Residual Fusion Network.
29. T2T-ViT [73]: Token-to-Token Vision Transformer.
30. VAN [74]: Visual Attention Network.
31. CSwin Transformer [75]: Cross-Stage win transformer.
32. DUCK-Net [17]: Deep Understanding Convolutional Kernel Network.
33. ST-UNet [76]: Spatiotemporal UNet.
34. SAM [77]: Segment Anything.
35. VM-UNet [78]: Vision Mamba UNet.
36. HC-Mamba [79]: Hybrid-convolution version of Vision Mamba.
37. EoMT [80]: Encoder-only Mask Transformer.
38. Med-SA [81]: Medical SAM Adapter.

We implemented both the Split and SplitFed versions of the networks marked with a green star in Figure 2. The following sections provide detailed descriptions of these implemented networks.

#### 2.3.1. UNet

**Figure 3:**
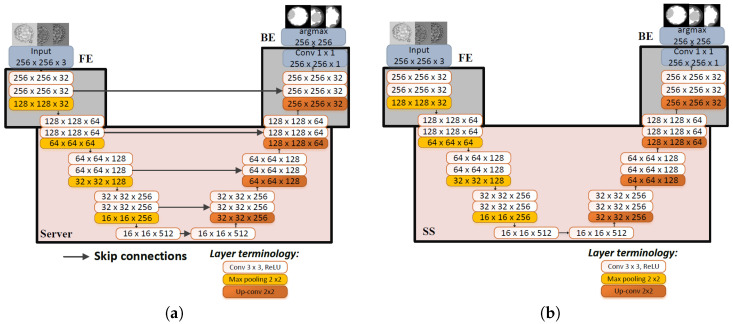
Split UNet and SegNet architectures. (**a**) Split UNet architecture. Ash-colored segments are the layers on the client side, and pink-colored segments are the layers on the server side. Note that this naming convention is applied to all other network figures in this paper. (**b**) Split SegNet architecture. A similar architecture to Split UNet without the skip connections.

UNet can be considered as the most well-known architecture specially designed for image segmentation tasks [10]. It embodies the encoder–decoder architecture with skip connections. UNet employs a process of gradually upsampling the features extracted by its encoder using transpose convolutions. By incorporating skip connections, UNet reaches greater depths than many other SoTA networks, resulting in significantly higher-quality outputs. Figure 3a shows the split version of the UNet used in this work.

#### 2.3.2. SegNet

The SegNet architecture shares significant similarities with UNet as it adheres to an encoder–decoder framework [11]. One of the distinguishing characteristics of SegNet from UNet is the absence of skip connections. Furthermore, SegNet diverges from the conventional upsampling operation found in many encoder–decoder architectures. Instead of traditional upsampling techniques, SegNet employs an approach referred to as “max unpooling”. This technique mitigates the need to learn how to upscale both the final output score and the feature maps from earlier layers. In a conventional encoder–decoder structure, such upscaling operations typically demand substantial learning. By integrating max unpooling, SegNet optimally addresses this challenge. Figure 3b depicts the split version of the SegNet used in our work.

#### 2.3.3. DeepLab

DeepLab was developed by Google [54] and has evolved into several versions, including DeepLab [54], DeepLabV1 [54], DeepLabV2 [55], DeepLabV3 [12] and DeepLabV3+ [13].

**Figure 4:**
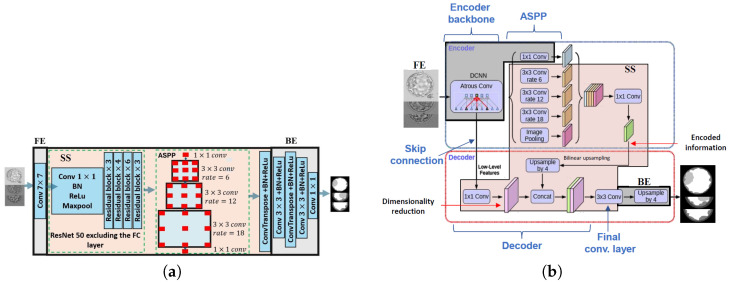
Split DeepLab architectures. (**a**) Split DeepLabV3 with ResNet50 as the backbone. (**b**) Split DeepLabV3+ architecture with the modified version of Xception as the backbone.

DeepLabV1 addresses those challenges presented by the SoTA networks, especially the FCN. It addressed the challenge of reduced feature resolution by employing atrous convolutions for upsampling. It addressed the challenge of reduced localization accuracy, due to DCNN invariance, by performing a post-processing procedure via conditional random fields (CRFs) [54]. DeepLabV2 addresses the challenge of handling objects at multiple scales by introducing the Atrous Spatial Pyramid Pooling (ASPP) method [55]. DeepLabV3 represents a significant advancement over DeepLabV2. Unlike DeepLabV2, which relies on the VGG16 backbone and a simpler ASPP module, DeepLabV3 introduces flexibility with diverse backbones like ResNet [82] and Xception [83]. The ASPP module is enhanced with parallel atrous convolutions, capturing multi-scale information more effectively. DeepLabV3 also incorporates global context through image-level features, distinguishing it from its predecessor. The subsequent DeepLabV3+ introduces additional efficiency with depthwise separable convolution. In summary, DeepLabV3 refines segmentation through advanced techniques, offering improved performance and adaptability over DeepLabV2 [12]. Figure 4a shows the split version of DeepLabV3 with ResNet50 as the backbone used in our work. DeepLabV3+ refines the segmentation output even further, especially along object boundaries. This is achieved using atrous separable convolutions in the encoder–decoder architecture and modifying the backbone version of the Xception network [83]. Figure 4b shows the split version of the DeepLabV3+ used in this work.

#### 2.3.4. RefineNet

**Figure 5:**
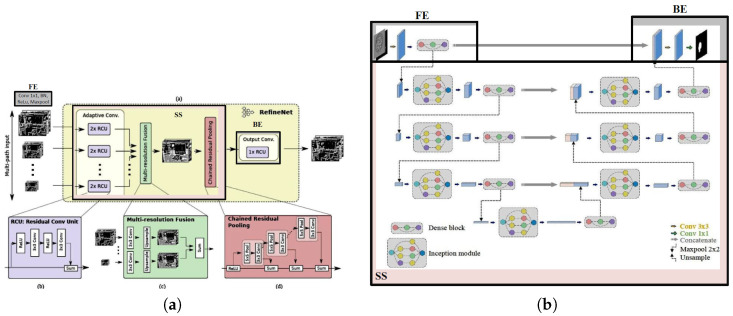
Split RefineNet and SUNet architectures. (**a**) Split RefineNet architecture. (**b**) Split SUNet architecture, including inception-dense modules in both encoder and decoder.

RefineNet is a generic multi-path refinement network that explicitly exploits all the information available along the down-sampling process to enable high-resolution prediction using long-range residual connections [14]. The authors highlighted limitations associated with typical CNNs, FCNs, and dilated convolutions. CNNs suffer from the downscaling of the feature maps, while FCNs output low-resolution predictions. Dilated convolutions are computationally expensive to train and could quickly reach memory limits. In RefineNet, fine-grained features from earlier convolutions are used directly to improve the deeper layers to capture high-level semantic features. This is called multi-path refinement. Chained residual pooling is also introduced by this work, where rich background contexts are captured in an efficient manner. Some variants of RefineNet are also proposed as a single RefineNet, 2-cascaded RefineNet, and 4-cascaded 2-scale RefineNet that upgrade its overall flexibility. Figure 5a shows the split version of RefineNet used in this work.

#### 2.3.5. SUNet

SUNet [16] is another U-shaped encoder–decoder network based on the inception module [84] and the dense block [85] to enhance the feature extraction and information reuse capabilities of the network. The idea behind the invention of SUNet is to make the standard UNet stronger in both width and depth. Four versions of SUNet are proposed as SUNet-V1, SUNet-V2, SUNet-V3, and SUNet-V4. As the version number increases, an improvement in classification and segmentation accuracy has been observed. It was originally invented as a federated brain tumor segmentation network. Figure 5b shows the structure of the split SUNet-V4 architecture.

#### 2.3.6. CGNet

**Figure 6:**
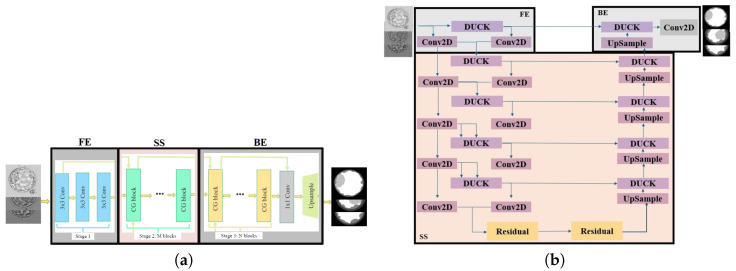
Split CGNet and DUCK-Net architectures. (**a**) Split CGNet architecture. (**b**) Split version of the DUCK-Net architecture.

CGNet is a recent network architecture designed for efficient and accurate semantic segmentation [15]. It stands out for having a lightweight architecture that benefits from context information to enhance segmentation performance. CGNet employs context blocks to capture contextual information at different scales, which helps the model to better understand the relationships between different objects and parts of an image. To increase the receptive field without adding to the computational overhead, CGNet utilizes dilated convolutions in the context blocks. Dilated convolutions allow the network to incorporate a larger context while keeping the number of parameters and computations low. The CGNet architecture uses feature fusion modules to combine features from different levels of the network. Such a fusion helps in integrating multi-scale information and improving segmentation performance. The network incorporates skip connections to help propagate information across different layers of the network. These connections enable better gradient flow during training and facilitate the overall optimization process. Despite being lightweight, CGNet delivers competitive performance on various semantic segmentation benchmarks, demonstrating its effectiveness in producing accurate segmentation results. Figure 6a shows the split version of CGNet used in our work.

#### 2.3.7. DUCK-Net

The DUCK-Net [17] uses an encoder–decoder architecture and has two main parts: an FCN block called DUCK that utilizes six different types of convolutional blocks at the same time, and a secondary UNet that keeps the low-level information intact. At each step, the DUCK block replaces the traditional pair of 3 × 3 convolutional blocks used in UNet. This allows the model to capture more details at each step while sacrificing finer, low-level information. Residual blocks are utilized in the last downsampling operation. Figure 6b shows the split version of DUCK-Net used in this work.

#### 2.3.8. Attention UNet

**Figure 7:**
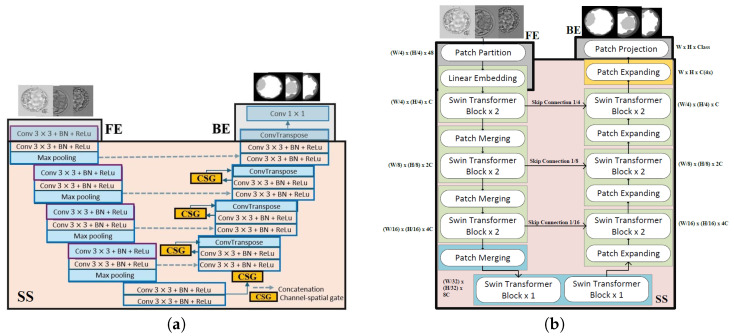
Split Attention UNet and Swin-UNet architectures. (**a**) Split Attention UNet architecture. (**b**) Split Swin-UNet architecture.

The Attention UNet, a modified UNet architecture for semantic image segmentation [18], integrates attention mechanisms [86] to enhance fine-grained detail capture and precise object localization. This attention mechanism enables the network to focus on informative regions while suppressing less relevant areas. Following an encoder–decoder structure, Attention UNet extracts hierarchical representations using the encoder and employs attention gates (channel-spatial gates) in the decoder. These gates assign weights to different regions in the feature maps, enhancing the focus on more important details during up-sampling. This attention-driven approach enhances the accuracy of segmentation masks by selectively emphasizing critical features, aiding in precise object and boundary localization. The split version of the Attention UNet used in our work is shown in Figure 7a.

#### 2.3.9. Swin-UNet

Swin-UNet is the first pure transformer-based U-shaped architecture [19], which combines the architectural advantages of the UNet and the Swin transformer [87]. This network includes the components of patch partition blocks, linear embedding blocks, Swin transformer blocks, patch merging blocks, patch expanding blocks, and linear projection blocks. Three skip connections are used to fuse the multi-scale features from the encoder with the upscaled features. Swin-UNet is famous for comparatively better performance than others due to its efficient attention mechanism, hierarchical feature representation, versatility, fewer parameters, and architectural design. Figure 7b shows the split version of the Swin-UNet used in this work.

### 2.4. Decision on Split Points Selection

In a SplitFed network, the selection of the most suitable split points is a crucial task. This choice of *where to split* the model is important to maintain the performance, communication, and overall efficiency of the SplitFed network [88]. While the ten architectures used in this study differ structurally, our split-point design follows a consistent principle: (i) the FE contains low-level feature extractors that interface with sensitive data, (ii) the SS contains the majority of parameters and computational load, and (iii) the BE handles prediction and gradients tied to GT labels to fully preserve the privacy. Although the proportion of layers varies across architectures, these design principles ensure functional consistency and reproducibility.

1. **Task-specific concerns:** The choice of split points is often guided by the nature of the machine learning task. For instance, in natural language processing, splits should occur at layers that capture semantic features, whereas in computer vision, splits should be at layers that capture high-level visual features.
2. **Communication constraints:** Split points should be strategically selected to minimize the overall computational load and communication costs associated with information transfer. This involves choosing points where computations are most intensive or sensitive, thus reducing overall latency and communication overhead.
3. **Model architecture:** Split points are selected at layers representing high-level features to enable clients to effectively learn task-specific details, ensuring that the model architecture supports the desired learning outcomes. Moreover, the edge blocks maintain the same dimensions, which is necessary for backpropagating gradients in the backward pass. Each sub-model generates its own gradients, making consistent dimensionality crucial.
4. **Privacy and security concerns:** To maintain data privacy, splits must be designed so that sensitive data remains on the client side. This approach involves creating two distinct model parts on the client side, with the front end handling sensitive data and the back end managing sensitive GTs.
5. **Computational capabilities of clients:** Split points are chosen to ensure that clients perform minimal computations, allowing those with limited resources to participate in the SplitFed training process without facing computational constraints.

In our experiments, we approached the determination of split points by evaluating each network individually. We carefully considered one or more of the criteria outlined above to ensure that the split points were chosen in a way that optimally addressed the specific characteristics and requirements of each network. This methodical approach allowed us to tailor our decisions to the unique aspects of each network, thereby enhancing the overall effectiveness of our experiments.

## 3. Experiments

In this section, we detail our experimental setup and results, followed by an evaluation that includes comparisons of performance and computational complexity.

### 3.1. Experimental Setup

We carried out the experiments with the three medical datasets introduced in Section 1:

- Blastocyst dataset [22]: includes 801 Blastocyst RGB images along with their GTs created for a multi-class embryo segmentation task. Each image is segmented into five classes: zona pellucida (ZP), trophectoderm (TE), blastocoel (BL), inner cell mass (ICM), and background (BG).
- HAM10K dataset [20]: The Human Against Machine dataset contains 10,015 dermatoscopic RGB images along with the corresponding binary GT masks, representing seven different types of skin lesions, including melanoma and benign conditions. Each image is segmented into two classes: skin lesion and background.
- KVASIR-SEG dataset [21]: contains 1000 annotated endoscopic RGB images of polyps from colonoscopy procedures, each paired with a binary GT segmentation mask. Each image is segmented into two classes: abnormal condition (such as a lesion, polyp, or ulcer) and background.

We randomly distributed samples from each dataset between clients. For the Blastocyst dataset, samples were assigned to four clients with 110, 90, 200, and 300 samples, respectively, with an additional 101 samples reserved for testing. The HAM10K dataset was divided among ten clients, receiving 1176, 588, 305, 941, 1058, 1294, 648, 942, 883, and 1132 samples each, with 1000 samples set aside for testing. The KVASIR-SEG dataset was partitioned into four clients with 125, 175, 275, and 325 samples, respectively, and 100 images were reserved for testing. In each segmentation task, 85% of the data was used for training and 15% for validation by each client.

All samples were resized to uniform dimensions of 240×240 pixels. Based on the network architecture and the dataset, we used several augmentation techniques to improve individual model training performance. Those include horizontal and vertical flipping, rotating, RGB shifting, normalizing, random brightness contrasting, etc. All experiments used the same loss function—*Soft Dice Loss* [89], Adam optimizer, and stopping criteria. We used network-specific and dataset-specific initial learning rates. *Intersection Over Union* (IoU) averaged over the sample size (average IoU), or equally the average Jaccard Index [90] was used as the performance metric. For the Blastocyst dataset, we utilized the average IoU of the TE, ZP, BL, and ICM components. Each centralized model was trained for 120 epochs. Each SplitFed model was trained for 10 global communication rounds, while each client trained their local models for 12 local epochs. The only difference between the three settings—centralized, locally centralized, and SplitFed is the training paradigm itself. This ensures a controlled comparison, ensuring fairness and transparency.

The training process begins with each client receiving randomly initialized copies of the FE and BE sub-models, along with a copy of the server sub-model. Clients then train these models locally for a fixed number of epochs, collaborating with their respective server sub-model copies. After local training, clients send the weights of their FE and BE sub-models to the server, where they are aggregated to form an updated global model. The updated sub-models are then distributed back to the clients for local validation, completing one global epoch. During training, clients send features from the FE sub-model to the server, which processes them and returns the output for BE processing. The BE sub-model generates predictions, computes the loss, and initiates back-propagation, with gradient updates passed from the BE sub-model to the server and then back to the FE sub-model.

Experiments were conducted on the Graham, Narval, and Cedar clusters using high-performance computing resources provided by Digital Research Alliance of Canada (https://ccdb.alliancecan.ca). We used a simple Linux utility for resource management scripts (SLURM) to request 1 GPU, 8 CPU cores, and 64 GB RAM per experiment on a single node. We have added the environment.yml file to the repository to support reproducibility. The codebase follows a standard PyTorch (v2.0.1) dataset/dataloader structure, allowing users to integrate their own datasets by implementing a dataset class consistent with the existing format. Actual deployments of the models are outside the scope of this initial release but will be included in future iterations of the repository. The initial release of the repository is available to download at https://vault.sfu.ca/index.php/s/ryhf6t12O0sobuX (password upon request to the authors).

### 3.2. Experimental Results

The following sections outline our experimental results. We show the results in two sections: Quantitative results and Qualitative results.

#### 3.2.1. Quantitative Results

We consider cases:

- **Centralized learning on full data:** We initially trained each network without splitting for image segmentation. We utilized the entire data from the three datasets separately. The average IoUs for all data samples in each set for the centralized models are displayed in the **C** column of Table 1.
- **Centralized learning locally at each client:** Secondly, we trained each client’s local data in a client-specific, centralized manner to ensure a fair comparison. In this step, each client trained the networks without data splitting. We recorded the IoUs for each client and computed the average, which is presented in the **L** column for each segmentation task in Table 1.
- **SplitFed learning:** Thirdly, we trained the SplitFed networks in collaboration with all clients. The IoUs of the SplitFed models are recorded in the **S** column for each segmentation task in Table 1.

#### 3.2.2. Qualitative Results

We included a visual comparison of three random samples from each test set during the SplitFed model training, as shown in Table 2.

### 3.3. Evaluation

#### 3.3.1. Testing Performance Comparison

When evaluating the cases described in Section 3.2.1, we expect the following IoU behaviors: Centralized learning on full data (**C**) should achieve the highest average IoUs due to access to the complete dataset, enabling better generalization. SplitFed learning should follow with slightly lower average IoUs, as collaboration among clients is effective but may face communication and synchronization challenges. Centralized learning locally (**L**) at each client is expected to yield the lowest average IoUs, as limited data at each client reduces generalization and segmentation performance. Table 1 confirms this: the highest average IoUs (of individual models) and the highest average over models are in the **C** column, followed by the **S** column, with the lowest in the **L** column. When looking at the average over the models (last row), the **C** column in the Blastocyst dataset showed 10.61% higher average IoU than the **L** column, and 1.26% higher average IoU than the **S** column. **C** column in the HAM10K dataset showed 5% higher average IoU than the **L** column, and 0.87% higher average IoU than the **S** column. **C** column in the KVASIR-SEG dataset showed 12.83% higher average IoU than the **L** column, and 2.01% higher average IoU than the **S** column.

Table 3 shows the three top-performing models for each case of the centralized, locally centralized, and SplitFed for the three segmentation tasks. As seen from the results, for smaller datasets like the Blastocyst dataset, models like Attention UNet and SUNet perform better during SplitFed, likely due to their ability to better generalize with limited data. Complex models like DeepLabV3 still perform well in both centralized and SplitFed, due to their pattern recognition capabilities. In the HAM10K dataset, which is much larger, more complex models such as CGNet and DeepLabV3 excel in the centralized case because they can better utilize the complete dataset for complex feature extraction. Even in SplitFed, models like DeepLabV3 and UNet continue to perform well, benefiting from efficient feature extraction suited to larger datasets. In the KVASIR-SEG dataset, models like DeepLabV3 and CGNet consistently perform well across all cases, showing their versatility. DUCK-Net also performs strongly in centralized cases, likely due to its efficiency in handling polyp segmentation tasks. However, in SplitFed cases, the Duck block at the edge layers appears to rely on tight synchronization, which may result in inefficiencies and slower convergence.

**Figure 8:**
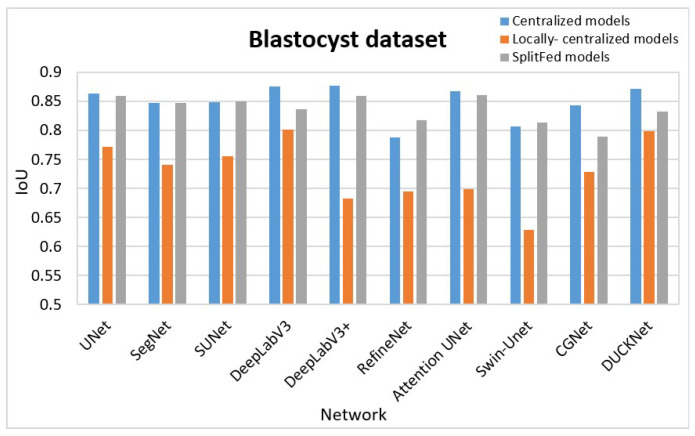
Comparison of results: Blastocyst Dataset. The blue-colored segment is applied to the centralized models; the orange-colored segment is applied to the locally centralized models; and the ash-colored segment is applied to the SplitFed models. Note that the same convention is applied in Figure 9 and Figure 10.

**Figure 9:**
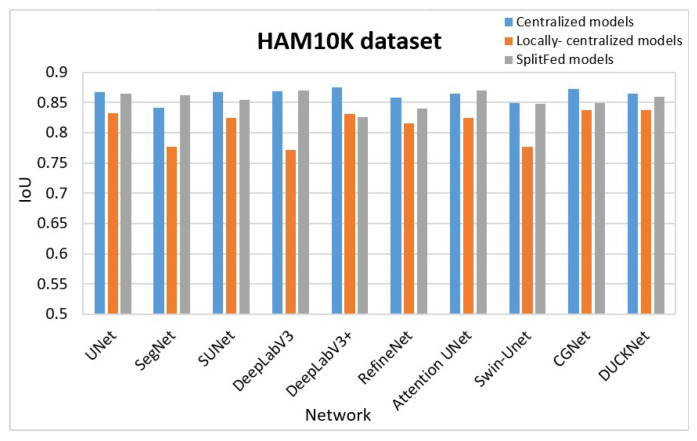
Comparison of results: HAM10K Dataset.

**Figure 10:**
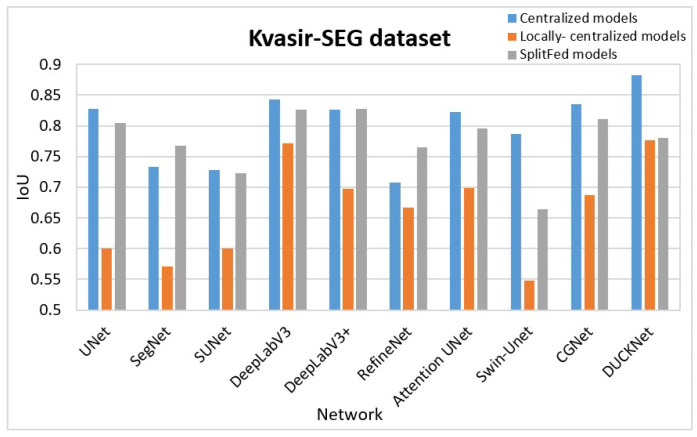
Comparison of results: KVASIR-SEG Dataset.

For the purpose of better comparison, we graphically represent the results in Table 1 in Figure 8, Figure 9 and Figure 10. Figure 8 summarizes the segmentation performance of various methods for the Blastocyst dataset, while Figure 9 shows the results for the HAM10K dataset. Comparisons for the KVASIR-SEG dataset are displayed in Figure 10. In these figures, the performance of the centralized model is represented in blue, the performance of the locally centralized models is depicted in orange, and the performance of the SplitFed models is illustrated in gray. From these figures, the SplitFed models have demonstrated superior performance compared to the locally centralized models, with performances closer to their corresponding centralized models.

#### 3.3.2. Comparison of Computational Complexity

We measured each model’s complexity with floating-point operations (FLOPs) by using the *ptflops* package in Python 3.9 [91]. Table 4 outlines the number of trainable parameters of each network, the measured FLOPs, the number of trainable parameters, and the FLOPs value with respect to UNet as the anchor. According to these metrics, CGNet has the least number of trainable parameters and the lowest FLOPs values compared to the other networks. RefineNet has the highest percentage of trainable parameters, while Attention UNet has the highest FLOPs value.

Further, Table 5 presents the layer proportions, trainable parameter distribution, and FLOPs for each architecture, highlighting how model complexity is partitioned across the FE, SS, and BE components in the SplitFed setup.

#### 3.3.3. Performance Comparison with Other Existing Methods

Table 6 presents the average IoUs of the other existing methods for semantic segmentation with the Blastocyst, HAM10K, and KVASIR-SEG datasets. For fairness and consistency, we only included studies that explicitly report average IoUs or average Jaccard Indices on the same datasets used in this work. Several mainstream segmentation models, such as SAM [77] do not provide reproducible public benchmarks or pretrained models for these datasets due to data availability, privacy restrictions, or data incompatibility. As a result, they cannot be reliably included in a quantitative comparison. In particular, the Blastocyst dataset is one of our proprietary datasets, so comparisons with SoTA are missing. Studies that solely mentioned accuracy metrics or any other metrics were intentionally omitted from the evaluation.

## 4. Limitations & Future Works

Throughout this work, we identified several limitations of the current MedSegNet10 release, as well as promising directions for further research. These are outlined as follows:

- First, our evaluation is limited to three distinct and commonly studied publicly available image types. Although we considered both multi-class (Blastocyst) and binary (HAM10K and KVASIR-SEG) segmentation datasets with varying sample sizes and styles to broaden the scope of generalization, these datasets may still not fully reflect the diversity of imaging characteristics, modalities, and annotation practices encountered in large-scale clinical deployments. Consequently, the reported results should not be regarded as definitive evidence of cross-domain generalizability. Future work will involve evaluating MedSegNet10 across a wider range of imaging modalities (e.g., CT, MRI, and multimodal acquisitions), annotation practices, and datasets to more comprehensively assess its robustness.
- Second, the datasets used in this study are modest in size. This limitation reflects the broader reality of medical imaging research- large open-source datasets are rare, expert-annotated data are costly, many image types are difficult to obtain, and privacy regulations restrict access. Consequently, it is naturally infeasible to evaluate decentralized frameworks on large-scale public datasets simply because such resources do not exist for many medical imaging tasks. MedSegNet10 should therefore be viewed as a foundational resource rather than a demonstration of large-scale scalability. Future work will involve expanding MedSegNet10 using larger institutional datasets and multi-centre cohorts, enabling more rigorous evaluation under realistic data volumes, acquisition variability, and deployment conditions.
- Third, the current experiments intentionally adopt IID data partitions to establish a controlled baseline and support benchmark reproducibility. This design choice does not reflect real clinical environments, where hospitals often exhibit strongly non-IID data distributions due to differing demographics, imaging devices, and annotation protocols. Non-IID robustness is a central challenge in federated learning, and evaluating MedSegNet10 under a range of realistic non-IID scenarios is an important direction for future work.
- Finally, recent capability-oriented reviews in smart healthcare [105] highlight how AI contributes to integrated monitoring [105,106], remote diagnostics [107], decentralized decision support [108], and data-driven hospital systems [109]. SplitFed architectures align naturally with these developments because they enable collaboration across institutions while preserving data privacy. Incorporating SplitFed networks from MedSegNet10 into smart-healthcare frameworks could facilitate interoperable, privacy-preserving segmentation tools that operate across hospitals or global imaging networks. This integration represents a promising avenue for extending MedSegNet10 beyond standalone model training towards deployment in real clinical infrastructures.

## 5. Conclusions

In this work, we introduced MedSegNet10, a new publicly accessible repository for medical image segmentation utilizing the SplitFed learning mechanism. Our main goal was to create a resource allowing researchers to easily integrate SplitFed models into their own applications without extensive customization by allowing the reuse of the networks in the MedSegNet10 repository. We designed, implemented, and optimized SplitFed versions of ten selected models to simplify the process and make the strengths of SplitFed accessible to a wider audience. In addition, we outlined identified limitations of the current MedSegNet10 release, with several future research directions in medical image segmentation that could benefit from SplitFed learning. MedSegNet10 provides a structured starting point for future work on SplitFed architectures, including extensions to more diverse datasets and modalities, non-IID clinical scenarios, and interpretability-oriented smart healthcare model designs.

## Figures and Tables

**Table 1:** Performance Comparison: Centralized (C), Locally Centralized (L), and SplitFed (S) Models (Average IoU metrics).

Model	Blastocyst Dataset			HAM10K Dataset			KVASIR-SEG Dataset		
	C	L	S	C	L	S	C	L	S
**UNet**	0.8643	0.7726	0.8593	0.8672	0.8320	0.8640	0.8271	0.6946	0.8042
**SegNet**	0.8475	0.7416	0.8475	0.8426	0.7773	0.8620	0.7337	0.5713	0.7669
**SUNet**	0.8487	0.7566	0.8504	0.8679	0.8241	0.8539	0.7280	0.6006	0.7233
**DeepLabV3**	0.8768	0.8016	0.8369	0.8699	0.7715	0.8696	0.8438	0.7715	0.8262
**DeepLabV3+**	0.8774	0.6834	0.8591	0.8715	0.8311	0.8262	0.8264	0.6965	0.8278
**RefineNet**	0.7881	0.6948	0.8181	0.8584	0.8161	0.8403	0.7083	0.6669	0.7652
**Attention UNet**	0.8673	0.6990	0.8605	0.8654	0.8241	0.8699	0.8236	0.6991	0.7961
**Swin-UNet**	0.8074	0.6283	0.8142	0.8492	0.7768	0.8478	0.7871	0.5483	0.6642
**CGNet**	0.8433	0.7287	0.7891	0.8728	0.8382	0.8490	0.8354	0.6868	0.8110
**DUCK-Net**	0.8725	0.7994	0.8321	0.8652	0.8389	0.8600	0.8824	0.7778	0.7800
**Average over models**	0.8493	0.7306	0.8367	0.8630	0.8130	0.8543	0.7996	0.6713	0.7765

**Table 2:** Qualitative comparison of three samples from SplitFed training.

Sample	Ground Truth	UNet	SegNet	SUNet	DeepLab V3	DeepLab V3+	RefineNet	Attention UNet	Swin-UNet	CGNet	DUCK-Net
**Blastocyst Dataset**											
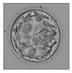 Blast_PCRM_R14-0411a.BMP	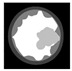	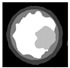	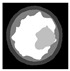	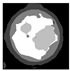	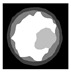	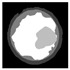	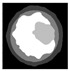	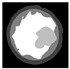	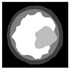	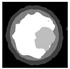	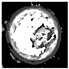
**HAM10K Dataset**											
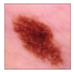 ISIC_0024308.jpg	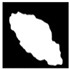	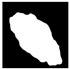	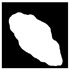	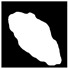	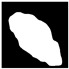	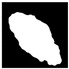	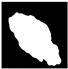	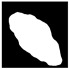	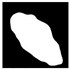	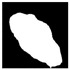	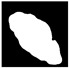
**KVASIR-SEG Dataset**											
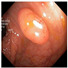 cju7bgnvb1sf808717qa799ir.jpg	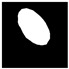	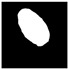	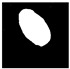	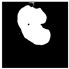	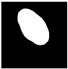	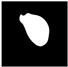	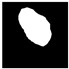	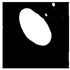	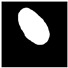	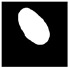	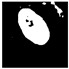

**Table 3:** Performance-wise comparison for SplitFed networks (Three top performing networks in each category are listed).

Model	Centralized Models	Locally Centralized Models	SplitFed Models
**Blastocyst dataset**	DeepLabV3+, DeepLabV3, DUCK-Net	DeepLabV3, DUCK-Net, CGNet	Attention UNet, DeepLabV3+, SUNet
**HAM10K dataset**	CGNet, DeepLabV3+, DeepLabV3	DUCK-Net, CGNet, DeepLabV3+	Attention UNet, DeepLabV3, UNet
**KVASIR-SEG dataset**	DUCK-Net, DeepLabV3, CGNet	DUCK-Net, DeepLabV3, DeepLabV3+	DeepLabV3+, DeepLabV3, CGNet

**Table 4:** Computational complexity-wise comparison (FLOPs are measured in “MAC”, which stands for “Multiply Accumulate”).

Model	Trainable Parameters (TPs)	FLOPs	TP as a %. of UNet	FLOPs as a %. of UNet
**UNet**	7.76M	10.52 GMAC	1%	1%
**SegNet**	9.44M	7.04 GMAC	1.22%	0.67%
**SUNet**	14.1M	24 GMAC	1.82%	2.28%
**DeepLabV3**	28.32M	12.83 GMAC	3.64%	1.21%
**DeepLabV3+**	54.70M	15.85 GMAC	7.05%	1.50%
**RefineNet**	118M	50.24 GMAC	15.20%	4.77%
**Attention UNet**	34.87M	51.03 GMAC	4.50%	4.85%
**Swin-UNet**	41.38M	8.67 GMAC	5.33%	0.82%
**CGNet**	0.30M	541.69 MMAC	0.039%	0.05%
**DUCK-Net**	22.67M	12.55 GMAC	2.92%	1.19%

**Table 5:** Comparison of layer proportions, trainable parameters (TPs), and FLOPs across FE, SS, and BE for the ten segmentation models.

Model	Layer Proportions			Trainable Parameters (TP)			FLOPs		
	FE	SS	BE	FE	SS	BE	FE	SS	BE
**UNet**	2 (8.0%)	21 (84.0%)	2 (8.0%)	0.002M (0.03%)	7.75M (99.85%)	0.009M (0.12%)	123.73 MMAC	13.09 GMAC	614.5 MMAC
**SegNet**	2 (6.25%)	28 (87.50%)	2 (6.25%)	0.001M (0.01%)	9.43M (99.90%)	0.009M (0.10%)	0.057 GMAC	8.46 GMAC	0.61 GMAC
**SUNet**	4 (6.78%)	52 (88.14%)	3 (5.08%)	0.006M (0.47%)	13.91M (98.72%)	0.11M (0.81%)	0.415 GMAC	38.817 GMAC	1.030 GMAC
**DeepLabV3**	1 (1.61%)	59 (95.16%)	2 (3.23%)	0.009M (0.03%)	28.24M (99.69%)	0.074M (0.26%)	0.31 GMAC	27.98 GMAC	0.77 GMAC
**DeepLabV3+**	1 (1.30%)	73 (94.81%)	3 (3.89%)	0.009M (0.02%)	54.403M (99.41%)	0.32M (0.57%)	0.19 GMAC	31.85 GMAC	0.49 GMAC
**RefineNet**	1 (0.71%)	138 (98.57%)	1 (0.71%)	0.009M (0.01%)	117.85M (99.95%)	0.012M (0.01%)	0.31 GMAC	59.84 GMAC	0.08 GMAC
**Attention UNet**	1 (2.63%)	35 (92.11%)	2 (5.26%)	0.35M (0.01%)	34.82M (99.88%)	0.009M (0.12%)	0.042 GMAC	13.10 GMAC	0.61 GMAC
**Swin-UNet**	2 (6.9%)	24 (82.8%)	3 (10.3%)	0.29M (0.7%)	39.23M (94.8%)	1.86M (4.5%)	0.08 GMAC	8.18 GMAC	0.42 GMAC
**CGNet**	3 (11.11%)	13 (48.15%)	11 (40.74%)	0.02M (6.69%)	0.06M (20.13%)	0.22M (73.18%)	0.32 GMAC	0.58 GMAC	0.66 GMAC
**DuckNet**	2 (6.67%)	26 (86.67%)	2 (6.67%)	0.11M (0.50%)	21.08M (93.00%)	1.47M (6.50%)	0.11 GMAC	11.55 GMAC	0.89 GMAC

**Table 6:** Performance comparison with other existing methods (NA: Not Applicable/Relevant experimental results are not reported or experiments have not been conducted in those studies).

SoTA Research	Centralized Models IoU	Federated Models IoU	SplitFed Models IoU
**Blastocyst Dataset**			
Our Previous research	0.798 (BLAST-NET [92]), 0.817 [88]	0.810 [88]	0.825 [88]
**HAM10K Dataset**			
FedPerl (Efficient-Net) [93]	0.769	0.747	NA
FedMix (UNet) [94]	NA	0.819 ± 1.7	NA
MALUNet [95]	0.802	NA	NA
FedZaCt (UNet) [96]	0.855	0.856	NA
FedZaCt (DeepLabV3+) [96]	0.861	0.863	NA
Chen et al. [97]	NA	0.892	NA
FedDTM (UNet) [98]	NA	0.7994	NA
**KVASIR-SEG Dataset**			
DUCK-Net [17]	0.9502	NA	NA
FCN-Transformer [99]	0.9220	NA	NA
MSRF-Net [72]	0.8508	NA	NA
PraNet [70]	0.7286	NA	NA
HRNetV2 [60]	0.8530	NA	NA
Subedi et al. [100]	0.81	0.823	NA
DilatedSegNet [101]	0.8957	NA	NA
DeepLabV3+ [101]	0.8837	NA	NA
Colonformer [102]	0.877	0.876	NA
SSFormer-S [103]	0.8743	NA	NA
SSFormer-L [103]	0.8905	NA	NA
FedDM [104]	0.5275 ± 0.0002	0.6877 ± 0.0308	NA

## Data Availability

This study only uses publicly available medical imaging datasets released with appropriate ethical approvals by their providers.
